# Genome-Wide Identification and Characterization of SQUAMOSA—Promoter-Binding Protein (SBP) Genes Involved in the Flowering Development of *Citrus Clementina*

**DOI:** 10.3390/biom9020066

**Published:** 2019-02-14

**Authors:** Ren-Fang Zeng, Jing-Jing Zhou, Sheng-Rui Liu, Zhi-Meng Gan, Jin-Zhi Zhang, Chun-Gen Hu

**Affiliations:** 1 Key Laboratory of Horticultural Plant Biology (Ministry of Education), College of Horticulture and Forestry Science, Huazhong Agricultural University, Wuhan 430070, China; Renfzeng@webmail.hzau.edu.cn (R.-F.Z.); hupodingxiangyu@mail.hzau.edu.cn (J.-J.Z.); zhimenggan@webmail.hzau.edu.cn (Z.-M.G.); chungen@mail.hzau.edu.cn (C.-G.H.); 2 State Key Laboratory of Tea Plant Biology and Utilization, Anhui Agricultural University, Hefei 230000, China; liushengrui@ahau.edu.cn

**Keywords:** *Citrus clementina*, SBP-box genes, gene expression, evolutionary comparison, functional diversity

## Abstract

SQUAMOSA-promoter binding protein (SBP)-box genes encode a family of plant-specific transcription factors that play vital roles in plant growth and development. In this study, 15 SBP-box genes were identified and isolated from *Citrus clementina* (*CclSBPs*), where 10 of these genes were predicted to be putative targets of *Citrus clementina* microRNA156 (CclmiR156). The 15 *CclSBP* genes could be classified into six groups based on phylogenetic analysis, diverse intron–exon structure, and motif prediction, similar to the *SQUAMOSA promoter binding protein-like (SPL)* gene family of *Populus trichocarpa* and *Arabidopsis thaliana*. Furthermore, *CclSBPs* classified into a group/subgroup have similar gene structures and conserved motifs, implying their functional redundancy. Tissue-specific expression analysis of *CclSBPs* demonstrated their diversified expression patterns. To further explore the potential role of *CclSBPs* during floral inductive water deficits, the dynamic changes of the 15 *CclSBPs* were investigated during floral inductive water deficits, and the results showed that some *CclSBPs* were associated with floral induction. Among these genes, *CclSBP6* was not homologous to the *Arabidopsis* SBP-box gene family, and *CclSBP7* was regulated by being alternatively spliced. Therefore, *CclSBP6* and *CclSBP7* were genetically transformed in *Arabidopsis*. Overexpression of the two genes changed the flowering time of *Arabidopsis*.

## 1. Introduction

SQUAMOSA-promoter binding protein (SBP)-box genes are a family of plant-specific transcript factors that play crucial roles in the regulation of plant growth and development [1]. The common feature of SPP genes is that their protein products contain a highly conserved SBP-box DNA-binding domain (approximately 76 amino acid residues). This domain features a zinc finger motif that contains two zinc finger domains. A putative nuclear localization signal (NLS) is located at the C-terminal of the SBP domain, which partly overlaps with the DNA-binding domain, particularly with the second zinc finger domain [2,3]. The SBP-box genes were first identified in snapdragon (*Antirrhinum majus* L.) [4], followed by other plants, such as *Arabidopsis thaliana* [5], silver birch (*Betula pendula*) [6], green alga (*Chlamydomonas reinhardtii*) [7], rice (*Oryza sativa*) [8], maize (*Zea mays*) [9], poplar (*Populus trichocarpa*) [10], tomato (*Solanum lycopersicum*) [11], apple (*Malus domestica*) [12], and grape (*Vitis vinifera*) [13]. In these plants, these genes were revealed to control flowering and fruit development, as well as other significant physiological processes.

A total of 16 SBP-box genes were identified in the *Arabidopsis* genome [5]. Ten members of the 16 *AtSPLs* including, *AtSPL2-6*, *AtSPL9-11*, *AtSPL13*, and *AtSPL15*, were targets of *miR156* [14,15,16,17]. *AtSPL3*, *AtSPL4* and *AtSPL5* contain complementary sequences of *miR156* in the 3′-UTR region, and they promote vegetative phase change and flowering [5,15,18]. *AtSPL2*, *AtSPL10*, and *AtSPL11* play important roles in regulating the morphological traits of leaves and flowers [19]. *AtSPL9* and *AtSPL15* are functionally redundant, and both of them can control the juvenile-to-adult phase transition and the leaf initiation rate [20]. in *Arabidopsis*, six SBP-box genes, including *AtSPL1*, *AtSPL7*, *AtSPL8*, *AtSPL12*, *AtSPL14*, and *AtSPL16*, are not targets of *miR156*. Among them, *AtSPL7* is involved in the regulation of copper homeostasis by controlling the expression of *COPT6* and its role in root and shoot development [21]. *AtSPL8* plays pivotal roles in regulating pollen sac development, male fertility, and Gibberellin (GA) biosynthesis and signaling [22,23,24]. *AtSPL14* regulates plant development and sensitivity to fumonisin B1 [25]. To date, the functions of *AtSPL1*, *AtSPL6*, *AtSPL12*, *AtSPL13*, and *AtSPL16* are still unknown, and further characterization is required to determine their function.

Currently, many members of the SBP-box family have been functionally characterized in plants other than *Arabidopsis*, and they have been shown to have diverse functions in plant growth and development. For instance, *BpSPL1* from *Betula pendula* was shown to be capable of binding a *cis*-element of *MADS5*, a close homolog of the *Arabidopsis FRUITFULL* gene, and this indicated a role for birch SPL genes in the regulation of flower development [6]. In the unicellular alga, an SBP-box protein from *Chlamydomonas reinhardtii* has been identified to regulate nutritional copper signaling [7]. In rice, *OsSPL14* regulated by *miR156* controls shoot branching in the vegetative phase [26]. Additionally, an SBP-box gene at the Colorless non-ripening (*Cnr*) locus from tomatoes is a pivotal factor in fruit ripening [27], while the maize *tasselsheath4* (*tsh4*) SBP-box gene regulates bract development and is important for branch meristem initiation and maintenance [28].

Citrus is economically one of the most important fruit crops in the world. To date, the analysis of SBP-box genes has focused mainly on the model plants, such as *Arabidopsis* and rice. However, little information is known about the evolution, functions, and expression patterns of the SBP-box genes in citrus compared with the model plants. Recently, *miR156-SPL* modules have been reported to regulate multiple biological processes in citrus, including juvenile-to-adult phase transition, drought stress induced flowering, fruit load, somatic embryogenesis, and starch accumulation of the callus cells [29,30,31,32]. In order to further elucidate the regulatory networks and functional characteristics of SBP-box genes during the process of growth and development in citrus, we identified 15 SBP-box genes from the citrus genome and further cloned these genes in this study, and then we performed a comprehensive analysis of the chromosomal location, phylogeny, gene structures, conserved motifs, and *miR156* target sites. The phylogenetic relationship and evolutionary comparison of SBP-box genes were also performed among various plants. Furthermore, the expression pattern of the 15 *CclSBPs* was studied in various tissues, as well as in the water deficit floral inductive condition. Moreover, transformation assays with two *CclSBP* genes suggested an important role in controlling the flowering of citrus. These results lay the foundation for further functional analyses of SBP-box genes in citrus and related species.

## 2. Materials and Methods

### 2.1. Plant Materials

The citrus samples were collected from the experiment fields at the National Citrus Breeding Center at Huazhong Agricultural University, Wuhan, China (30° 28′ N, 114° 21′ E, 30). In this study, we used 14-year-old Clementine mandarin trees (*Citrus clementina* cv. Monreal) propagated by bud grafting to trifoliate orange rootstocks (*Citrus trifoliata*), where a total of 9 adult healthy trees were selected and randomly divided into three groups with three plants per group. All the samples were collected from three groups of trees and used as biological repeats. For spatial expression analysis of the *CclSBPs*, the samples were collected and total RNA was isolated from healthy and mature leaves, flowers at full bloom, young fruits at 90 days after flowering, mature fruit at the picking period, and roots of *C. clementina*, respectively. The tissues were named Rt (roots), Sm (stems), Lf (leaves), Pe (petal), St (stamen), Ca (carpel), Re (receptacle), YF (young fruits), YS (young seeds), MF (mature fruits), and MS (mature seeds). All plant tissues were sampled according to the demands of each experiment, and they were immediately frozen in liquid nitrogen and stored at −80 °C until used.

### 2.2. Identification and Molecular Cloning of CclSBPs

The nucleotide and deduced amino acid sequences of 16 SPL genes from *Arabidopsis* [5] were obtained from TAIR (The *Arabidopsis* Information Resource) database [33]. A genome-wide search of *CclSBP* genes was carried out using Basic Local Alignment Search Tool (BLAST) analyses, with the 16 *AtSPLs* genes used as queries against the *C. clementina* genome [34]. All non-redundant protein sequences of the putative citrus SBP-box genes were checked for the SBP domain using the protein families database (Pfam) [35]. To verify the coding regions of *CclSBPs*, gene-specific primers were designed for amplification of the 15 *CclSBP* genes using polymerase chain reaction (PCR) with cDNA templates from leaves of *C. clementina* (Supplementary Table S1). The PCR was performed in a 20 µl system, in a Veriti 96-Well Thermal Cycler. Amplified products were separated in a 1.0% agarose gel electrophoresis. The target bands were recovered, cloned into pMD18-T vectors (TaKaRa Biotech, Dalian, China), and then transformed into the *Escherichia coli* strain DH5a. Three positive clones were sequenced for each candidate SBP-box gene.

### 2.3. Phylogenetic Analysis of CclSBPs

Multiple alignments of the SBP-box protein sequences were performed using the ClustalW program [36]. The sequence logo was obtained using the online Weblogo platform [37]. The phylogenetic trees were generated by MEGA 6.0, using the maximum likelihood (ML) algorithm [38]. Bootstrap analysis with 1000 replicates was used to evaluate the significance of the nodes. Jones–Taylor–Thornton (JTT) model was used to ensure that the divergent domains could contribute to the topology of the ML tree [38].

### 2.4. Gene Structure, Conserved Domain and miR156 Target Site Analysis of CclSBPs

The exon/intron organization of *CclSBPs* was determined by comparing the coding sequences to their corresponding genomic sequences using the gene structure display server (GSDS) program [39]. The simple modular architecture research tool (SMART) and multiple em for motif elicitation (MEME) were used to identify the conserved motif structures of the SBP-box protein sequences [40,41]. The sequence target of *miR156* (*miR156a* and *miR156b*) was determined in a previous work on *C. trifoliata* [42]. The targets of *miR156* were predicted by searching the coding regions, as well as the 3′-UTR of all the *CclSBP* genes, using the psRNATarget tool with default parameters as in Reference [43].

### 2.5. Identification of the Specific Repetitive Elements of CclSBPs

To determine whether specific repetitive elements drive the sequence divergence of *CclSBPs*, the tandem repeat (TR), transposable element (TE), low-complexity repeat (LCR), and simple sequence repeat (SSR) were investigated in the region that was 1.5 kb upstream of the 5′ UTR to the 3’-UTR of the genes. TRs were searched using the Tandem Repeat Finder 4.04 [44], with default parameter values, respectively. TEs and LCRs were detected using RepeatMasker [45]. SSRs were examined using the web-based software SSRIT [46]. Putative *cis*-elements in the promoter regions of *CclSBPs* were annotated using the PlantCARE software [47]. The motifs putatively involved in plant growth and development, hormone responses, light response, and stress responses were summarized in this study.

### 2.6. Co-Expression Analysis of CclSBPs During Floral Inductive Water Deficits

The published transcriptome from the lemon bud provided a useful complement to understand the expression patterns of the *CclSBPs* during floral inductive water deficits [32]. The co-expression network of the *CclSBPs* was constructed, the correlations between two genes above 0.85 were kept, and then visualized in Cytoscape as in Reference [48]. The Gene Ontology (GO) and Kyoto Encyclopedia of Genes and Genomes (KEGG) analyses of genes in the co-expression network were conducted in R, using the packages “clusterProfiler” and “pathview” as described in reference [49,50]. The predicted protein interaction network of *CclSBPs* proteins in citrus was built according to knowledge obtained from *Arabidopsis*.

### 2.7. Real-Time Polymerase Chain Reaction (RT-PCR)

Total RNA was isolated using the Oligotex mRNA mini kit (Qiagen, Gaithersburg, MD, USA), according to the manufacturer’s instructions. The RNA preparation was treated with DNase I (Promega, Madison, WI, USA), and first strand synthesis of the cDNA was performed using RT Primer Mix and Primescript RT Enzyme Mix I (life technology). Three biological replicates were performed in this study. To normalize the variance among samples, the expression level of the citrus *β-actin* was used as the internal control. The program was performed as described previously in [51]. The gene-specific primers for real-time polymerase chain reaction (RT-PCR) of *CclSBPs* were listed in Table S1.

### 2.8. Arabidopsis Transformation

The full-length cDNA sequences were ligated into pBI121 driven by CaMV35S, and then transferred into the Agrobacterium strain GV3101. *Arabidopsis* (Col) was transformed using the floral dip method as in Reference [52]. The third generation of transgenic lines and wild type were grown under long-day conditions. Plants were arranged in a randomized complete block design with three blocks. Each genotype within a block was represented by five plants. Thus, 15 plants from each genotype were observed. Flowering time was measured by counting the number of rosette leaves and the number of days from sowing to when the first flower bud was seen. Statistical analysis was performed by one-way analysis of variance (ANOVA), taking *p* < 0.05 as significant.

## 3. Results

### 3.1. Identification, Cloning and Sequence Feature Analysis of CclSBPs

**Figure 1:**
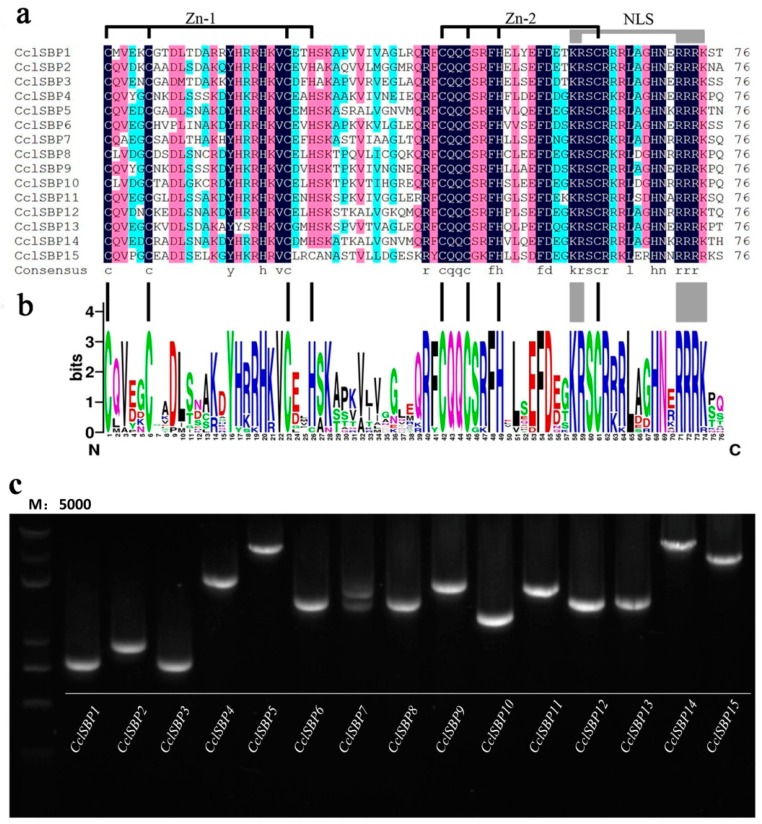
SBP-domain alignment of the *CclSBPs* and polymerase chain reaction (PCR) cloning. (**a**) Multiple alignment of the SBP domains from the citrus SBP-box protein based on ClustalW program, and the two conserved zinc finger structures (Zn-1, Zn-2) and nuclear localization signal (NLS) are demonstrated; (**b**) sequence logo of the citrus SBP domain. The overall height of each stack represents the degree of conservation at this position, while the height of the letters within each stack indicates the relative frequency of the corresponding amino acids; (**c**) Detection of 15 the *CclSBP* family members in *Citrus clementina*. Lane 1: M, molecular marker, Marker 5000; From Lane 2 to Lane 16 is *CclSBP1*, *CclSBP2*, *CclSBP3*, *CclSBP4*, *CclSBP5*, *CclSBP6*, *CclSBP7*, *CclSBP8*, *CclSBP9*, *CclSBP10 CclSBP11*, *CclSBP12*, *CclSBP13*, *CclSBP14*, and *CclSBP15*, respectively.

A total of 15 unique genes containing SBP motif were indentified after removing the redundant sequences in this study and they were assigned names from *CclSBP1* to *CclSBP15* based on their location on seven scaffolds (equivalent to seven chromosomes). The length of the *CclSBPs* varied from 1247 (*CclSBP1*) to 7069 bp (*CclSBP15*), and the length of the coding sequences varied from 393 (*CclSBP1*) to 3309 bp (*CclSBP12*). Furthermore, the number of transcripts of each *CclSBP* gene and the percentage of the expressed sequence tags (EST) that match with the database are shown in Table 1. *CclSBPs* were unevenly distributed across seven chromosomes: chromosomes 3 and 4 displayed four genes, chromosomes 2 and 9 had three genes, and chromosomes 1, 5, and 6 only showed one gene (Table 1). There was only one conserved domain (the SBP domain) found to be shared by all the *CclSBPs*. The SBP domains of the *CclSBPs* were very similar, with a high conservation at certain positions (Figure 1a). All of the *CclSBPs* shared two zinc finger-like structures (Zn-1, Zn-2) and a highly conserved bipartite nuclear localization signal (NLS). For all the *CclSBPs*, the second zinc finger within the SBP domain was CysCysHisCys, whilst the first zinc finger was CysCysCysHis, except for the *CclSBP15*, where the other zinc finger type was CysCysCysCys (Figure 1b). Remarkably, the second zinc finger-like structure of all the *CclSBPs* was partly overlapped with the nuclear localization signal.

To further verify the results of the *CclSBP* gene models obtained from computational prediction, we cloned and sequenced the coding region of all the *CclSBP* (Figure 1c). For most *CclSBP* genes, clear single bands were amplified, except *CclSBP7*. Three positive cloning were sequenced for each gene, and the complete cDNA of each gene was submitted to the GenBank (KT601172–KT601186). Subsequently, the alignment between these cloned cDNA and the coding sequences from the *C. clementina* genome database were investigated, and the results showed that no differences were found in eight *CclSBPs* (*CclSBP2*, *CclSBP4*, *CclSBP5*, *CclSBP6*, *CclSBP7*, *CclSBP8*, *CclSBP10*, and *CclSBP12*). Only one single nucleotide polymorphism (SNP) site was found in *CclSBP1*, *CclSBP3*, *CclSBP11*, and *CclSBP13*, respectively; three SNP sites were found in *CclSBP15*; 36 SNP sites were found in *CclSBP14*; and one 15 bp deletion was found in *CclSBP9*.

### 3.2. Gene Structure, Conserved Domain and miR156 Target Site Analysis of CclSBPs

**Figure 2:**
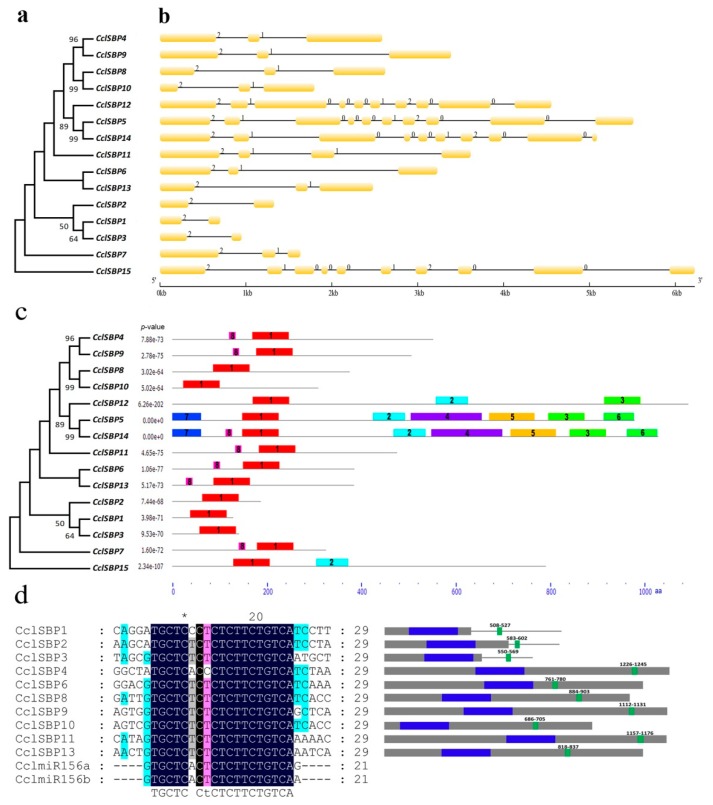
Phylogenetic tree, gene structures and conserved motifs of the *CclSBPs*. (**a**) Phylogenetic tree was constructed based on 15 citrus SBP-box protein sequences; (**b**) exon-intron structures of the *CclSBPs*. Introns are represented by lines. Exons are indicated by yellow boxes; (**c**) distribution of the conserved motifs in the *CclSBPs*. Motifs represented with boxes are predicted using Multiple Em for Motif Elicitation (MEME). The number in the boxes (1–8) represents motif 1–motif 8, respectively. Box size indicates the length of the motifs; (**d**) Multiple alignment of the CclmiR156 complementary sequence with 10 *CclSBPs* and their binding site position.

To provide further insights into the evolutionary relationship of *CclSBPs*, a phylogenetic tree was constructed based on the citrus full-length SBP-box protein sequences. The 15 SBP-box genes were clustered into different groups based on the evolutionary relationship (Figure 2a). The exon/intron organization in the coding sequences of each *CclSBP* gene was also performed, and the number of introns in the coding region of 15 *CclSBPs* varied from 1 to 9 (Figure 2b). Most closely related members shared similar exon/intron structures in terms of intron number and exon length. In addition to the SBP domain, other conserved motifs could also be important for the functioning of SBP-box proteins. Hence, we further searched the conserved motifs using the Multiple Em for Motif Elicitation (MEME) online server and we applied an e-value cut-off of 10^−10^ to the recognition. As a result, eight conserved motifs were discovered among the 15 *CclSBPs* proteins (Figure 2c). The number of motifs in each *CclSBPs* varied from 1 to 8 (Figure 2c). Motif 1 was actually the SBP domain and it existed in all the *CclSBPs*. However, the putative functions of the other motifs are currently unknown and they need to be further investigated. As expected, most of the closely related members had common motif compositions, such as *CclSBP4* and *CclSBP9*, *CclSBP8* and *CclSBP10*, *CclSBP5* and *CclSBP14*, *CclSBP1* and *CclSBP3*. This probably implied functional similarities in plant growth and development amongst these *CclSBPs* which shared similar gene structures and conserved motifs.

To understand the *miR156*-mediated posttranscriptional regulation of *CclSBPs*, a total of 10 *CclSBPs* were predicted to be targets of *miR156* (Figure 2d). The target sites of *miR156* were located in the coding regions for five *CclSBPs* (*CclSBP4*, *CclSBP6*, *CclSBP9*, *CclSBP11*, and *CclSBP13*) and two *CclSBPs* (*CclSBP8* and *CclSBP10*) belonging to two different subgroups. The target sites for the other three *CclSBPs* (*CclSBP1*, *CclSBP2*, and *CclSBP3*) belonging to a same subgroup, were located in the 3’-UTR regin. It suggested that *miR156*-mediated posttranscriptional regulation of SBP-box genes was conserved in these plant species.

### 3.3. Comparative and Phylogenetic Analysis of the SBP-Box Genes in Various Plants

**Figure 3:**
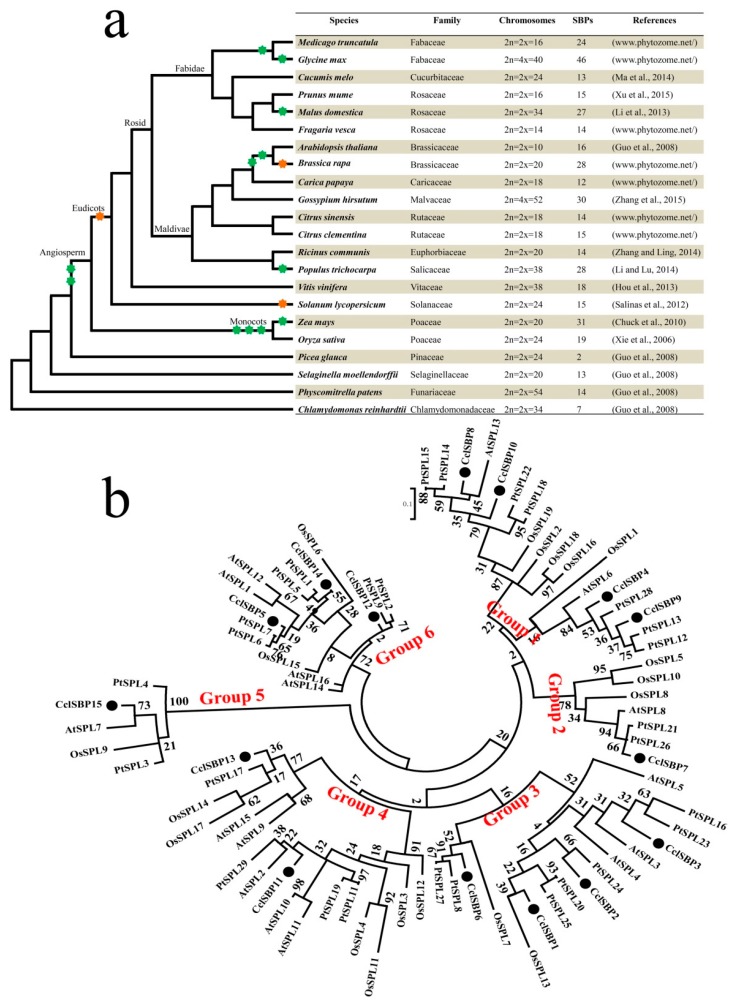
Comparative and phylogenetic analysis of the SBP genes in various plants. (**a**) The species tree and number of SBP-box paralogs in each species. The species tree and the duplication events are based on information in phytozome and plant genome duplication database (PGDD). The green star represents whole genome duplication and the yellow star represents whole genome triplication, respectively. Branch lengths do not represent time or the relative amount of character change; (**b**) phylogenetic analysis of citrus and other plant SBP-box genes. Phylogenetic tree was constructed using SBP domain protein sequences from citrus, *Populus* (*PtSPLs*), *Arabidopsis* (*AtSPLs*), and rice (*OsSPLs*), using MEGA 6.0 software [38].

Previous studies and a growing number of fully sequenced plant genomes make it possible to perform a comparative genomic analysis of the SBP-box gene family across a wide range of plant species. In this study, we analyzed some of the major types of model organisms whose genomes have already been sequenced, including green algae, chlorophytes, moss, lycophyte, eudicots and monocots (Figure 3a). The number of SBP-box genes has been identified in 15 plant species, and the SBP genes of seven plant species from the Phytozome database (*Medicago truncatula*, *Glycine max*, *Fragaria vesca*, *Brassica rapa*, *Carica papaya*, *Citrus sinensis* and *C. clementina*) were checked in our study. We constructed a phylogenetic tree and displayed the duplication events of these 22 species (Figure 3a). In brief, the number of SBP-box genes in algal, lowland species, monocots, and dicots offered further insight into the evolutionary processes of this gene family.

On the other hand, to further investigate the evolutionary relationship between citrus, *Arabidopsis*, poplar and rice, a total of 79 SBP-box genes were used for phylogenetic analysis based on their conserved SBP domains (Figure 3b). These SBP genes were also clustered into six groups, each of which contained at least one *AtSPL* and one *CclSBP*. It was shown that the number of citrus, *Arabidopsis*, *Populus* and rice SBP genes varied across the six groups (the *CclSBPs* in group 1 to group 6 had 4, 1, 4, 2, 1, 3 members, respectively; the *PtSPLs* in group 1 to group 6 had 7, 2, 7, 4, 2, 6 members, respectively; the *OsSPLs* in group 1 to group 6 had 5, 3, 2, 6, 1, 2 members, respectively; and the *AtSPLs* in group 1 to group 6 had 2, 1, 3, 5, 1, 4 members, respectively). Interestingly, the SBP domain of SBP-box genes in group 5 were divergent from the other groups (Figure 3b). The N-terminal zinc finger of group 5 SBP-box genes has four cysteine residues in the SBP domain, while SBP-box genes in other groups mainly contain three cysteines and one histidine. Based on the phylogenetic tree, the miR156-targeted SBP-box genes, including sequences from rice, *Arabidopsis* and poplar, were distributed into only three of the subgroups (Groups 1, 3 and 4). Generally, most of the *CclSBPs* showed a closer phylogenetic relationship with *PtSPLs* than *AtSPLs*, and they showed the most distant phylogenetic relationship with *OsSPLs*. These results indicated that orthologous genes between woody plants showed higher similarities than those genes between wood plants and herbaceous plants, and orthologous genes between dicots had a higher similarity than between monocots and dicots. Additionally, it is worth noting that the clusters group together *Arabidopsis*, rice, poplar, and citrus genes, which are closer to their orthologous counterparts from the other species than to the other family members from their own species. These results indicated that the SBP-box family of genes was present in the ancestor plants that gave rise to the monocot and dicot lineages, making it possible to estimate the minimum number of SBP-box genes in this ancestor.

### 3.4. Specific Repetitive Elements in the CclSBPs

**Figure 4:**
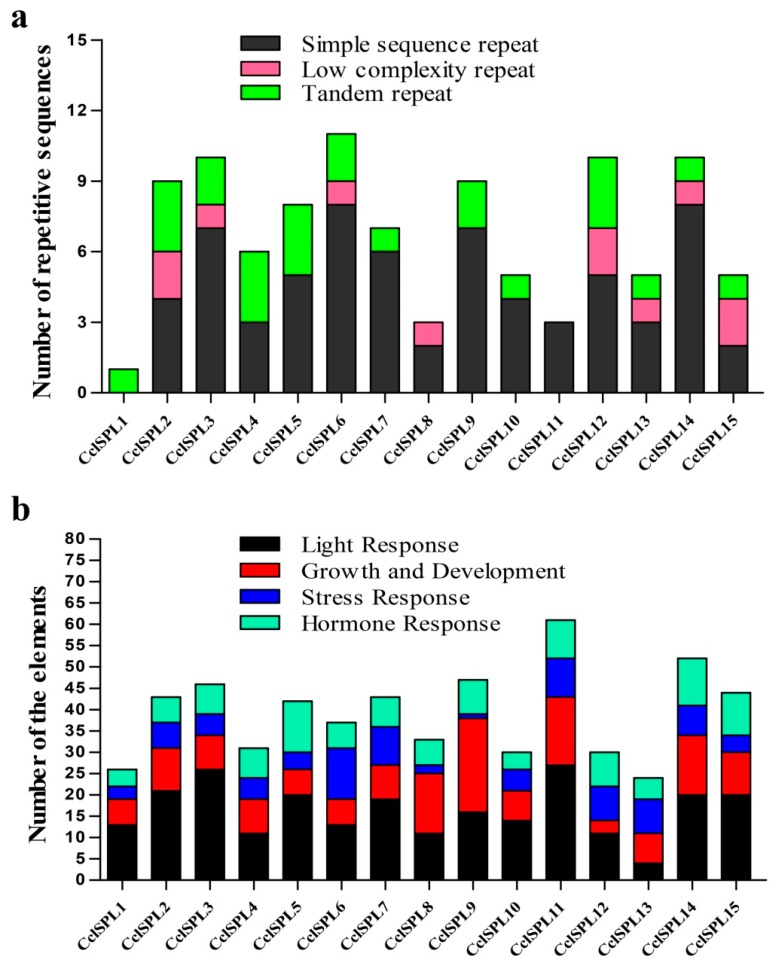
The number statistics repetitive sequences of the full-length sequence and *Cis*-elements in the promoter regions of *CclSBPs*. (**a**) The number of repetitive sequences was counted for every 1.5 kb of promoter and DNA. (**b**) Elements were identified in the 1.5 kb sequences upstream of the start codon of the *CclSBP* genes using the PlantCARE database. The elements associated with specific functions are indicated by different colors for each gene.

To characterize the sequence divergence of *CclSBPs*, the spatial distribution of repetitive sequences with respect to the genomic position of the *CclSBP* was also examined. We investigated the distribution of four types of repetitive sequences that are frequently found in the promoter and coding regions of the *CclSBP* (TR, TE, LCR, and SSR). The results indicated that all the *CclSBP* had repetitive sequence insertions and the repetitive sequences were frequent in the promoter and genome DNA regions (Figure 4a). Different types of repetitive sequences frequency were different in the 15 *CclSBP* genes, and SSR had the largest number and was present in all the *CclSBPs* except *CclSBP1*. TR were also found to be present in the genome regions of all the *CclSBPs*, except *CclSBP8* and *CclSBP11*. LCR was only found in eight *CclSBP* genes. It is worth noting that TE was not found in any of the *CclSBP* sequences (Figure 4a).

*Cis*-elements play important roles in the regulation of gene transcription during plant growth, development, and stress responses. To understand the transcriptional regulation mechanisms, the *cis*-elements in the promoter regions of *CclSBP* genes were identified through the PlantCARE database (Figure 4b). Except for the common *cis*-acting elements, such as CAAT-box and TATA-box, many *cis*-elements were identified in the promoter regions of the 15 *CclSBP* genes (Table S2). According to their putative functions, these elements were categorized into four classes. The results showed that light-responsive elements had the largest number and were present in all the promoter regions of *CclSBP*. The hormone responsive elements, plant growth and development, and stress responsive elements were also found to be present in the promoter regions of all the *CclSBP* genes. In addition, other rarely distributed *cis*-elements in *CclSBP* were also found to be functionally involved in transcription regulation, circadian control, protein binding, and stress responsiveness. Therefore, the transcription of the *CclSBP* genes could be regulated by various environmental and developmental changes, which implied that *CclSBP* genes were involved in important physiological processes and developmental events. Furthermore, limited similar *cis*-elements distribution was observed amongst these *CclSBP* genes, even for those *CclSBP* in the same phylogenetic group.

### 3.5. Analysis of the Expression Patterns of the CclSBPs Gene Family

**Figure 5:**
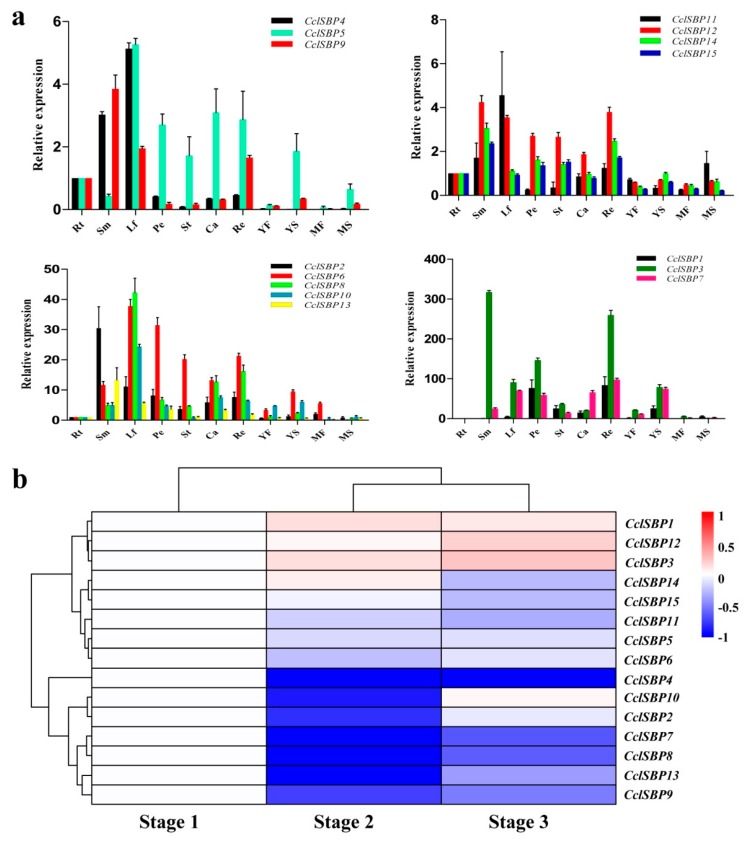
Analysis of the expression patterns of the *CclSBPs* gene family. (**a**) Relative quantities of *CclSBPs* in various tissues. Fold changes of the transcript levels in the root (Rt), stem (Sm), leaf (Lf), petal (Pe), stamen (St), carpel (Ca), receptacle (Re), young fruits (YF), young seeds (YS), mature fruits (MF), mature seeds (MS) of citrus are shown. Error bars represent the standard deviations of mean value from at least three biological replicates. (**b**) Cluster analysis of the expression profiles of *CclSBPs* at three stages (stage 1: one week before water deficit; stage 2: one week after the beginning of water deficit; and stage 3: one week after release from the water deficit), as shown in a previous study [32]. Each column represents a sample, and each row represents a single citrus transcript sequence. The bar represented the scale of relative expression levels of differentially expressed genes (DEGs), and the colors indicate relative signal intensities.

To preliminarily elucidate the roles of *CclSBPs* in citrus growth and development, we examined the relative expression levels of 15 *CclSBPs* in 11 different tissues (Figure 5a). All the *CclSBPs* were detected in at least one of the tissues examined, but a differential expression was observed. The expression data showed a high variability in the transcript abundance of *CclSBPs* in various tissues and organs, strongly indicating the diversified functions of *CclSBPs* in citrus growth and development. It is worth noting that many genes have shown similar expression patterns, such as *CclSBP4* and *CclSBP9*, *CclSBP8* and *CclSBP10*, *CclSBP12* and *CclSBP14*, and *CclSBP2* and *CclSBP3* belonging to the same subgroup (Figure 2a), indicating their redundant functions. Nevertheless, the expression patterns of a few similar gene pairs, including *CclSBP1* and *CclSBP3*, *CclSBP6* and *CclSBP13*, and *CclSBP5* and *CclSBP14* belonging to the same subgroup (Figure 2a) are distinct. This suggests that these genes may play different roles in citrus growth and development, although they have high sequence similarity.

To explore the potential role of *CclSBPs* during floral inductive water deficits, RNA sequencing was performed in a previous study on lemon buds at three stages (Stage 1: one week before water deficit; Stage 2: one week after the beginning of water deficit; and Stage 3: one week after release from water deficit) [32]. We classified the 15 *CclSBPs* into four clusters based on the similarity of the expression patterns (Figure 5b). Cluster 1 genes (including *CclSBP3* and *CclSBP12*) were induced immediately at stage 1 and mostly maintained high expression levels at Stage 3(Figure 5b). These genes were significantly induced at the beginning of the water deficit, indicating that the gene cluster might play a key role in the necessary growth and development of citrus. Cluster 2 genes (including *CclSBP11*, and *CclSBP15*) were suppressed immediately at stage 1 and mostly maintained low expression levels at Stage 3 (Figure 5b). Cluster 3 genes (including *CclSBP2*, *CclSBP6*, *CclSBP7*, *CclSBP8*, *CclSBP9*, *CclSBP10*, *CclSBP12*, and *CclSBP13*) were transiently suppressed at stage 2 and were then induced at stage 3 (Figure 5b). This cluster showed up-regulated expression at later stages of treatment, indicating the expression of genes involved in the flowering and recovery of vegetative growth. Cluster 4 genes (including *CclSBP1*, *CclSBP5*, and *CclSBP14*) were transiently induced at stage 2 and were then suppressed at stage 3 (Figure 5b). The suppression of the gene cluster may have implied possible involvement in the drought stress response, floral induction, and flower bud differentiation of lemon. A total of four genes (*CclSBP4*, *CclSBP7*, *CclSBP8*, and *CclSBP13*) were considered to be differentially expressed based on a probability ≥0.8 and an absolute value of log_2_^Ratio^ ≥ 1 as a threshold. These findings suggested that these genes may play important roles during the floral inductive water deficit process.

### 3.6. Co-Expression Analysis of the CclSBPs Under Floral Inductive Water Deficits Conditions

**Figure 6:**
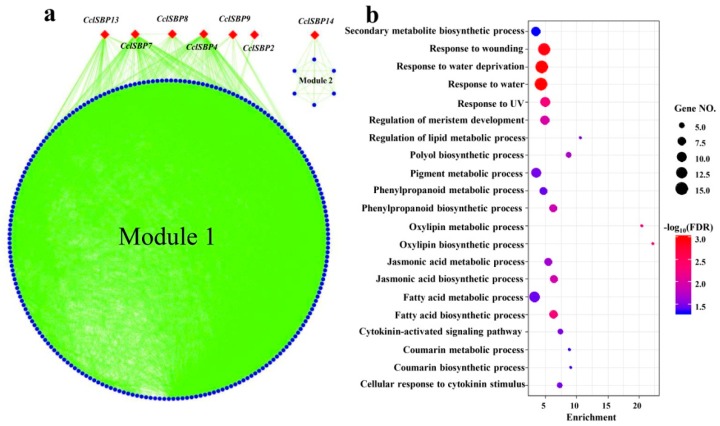
Co-expression network analysis of the *CclSBPs* using the data from a previous study [32]. (**a**) *CclSBPs* centered the gene co-expression network under floral inductive water deficit conditions. (**b**) The biological processes of the GO term that were significantly enriched in the *CsSBP* centered network.

To uncover the possible roles that *CclSBPs* played in citrus, a co-expression analysis was performed under the floral inductive water deficit conditions. There were 15 *CclSBPs* together with the 1638 differentially expressed genes (DEGs) forming a co-expression network via 20,450 interactions (edges). The co-expression network naturally clustered into two modules with certain *CclSBPs* (Figure 6a). For instance, module 1 represented the largest module in the co-expression network containing six *CclSBPs* (including *CclSBP2*, *CclSBP4*, *CclSBP7*, *CclSBP8*, *CclSBP9*, and *CclSBP13*). Genes from module 2 were uniquely co-expressed with *CclSBP14*. Gene ontology (GO) term analysis of the *CclSBPs* centered co-expression network uncovered the possible roles of *CclSBPs* in the processes of growth and stress response (Figure 6b). For instance, the genes from the co-expression network were enriched in terms of the “response to water/water deprivation”, “response to wounding”, “regulation of meristem development”, “photosynthesis”, as well as in terms of hormone synthesis and metabolism (Figure 6b). As mentioned above, the co-expression network was naturally separated into two dependent modules; and it was interesting to check the specific roles enacted by the different modules. The GO term analysis further confirmed that the genes from the different modules had distinct functions involved in the different aspects of plant growth and development.

### 3.7. Phenotypes of CclSBP6 Over-Expression in Arabidopsis

**Figure 7:**
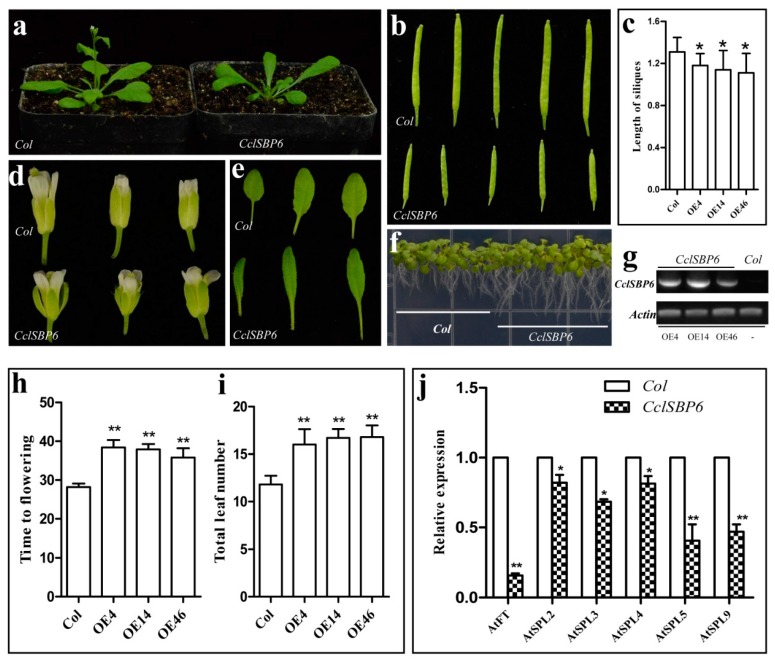
The phenotypes of transgenic *Arabidopsis* with *CclSBP6* under long-day conditions. (**a**) Phenotypes of transgenic *Arabidopsis* with *CclSBP6*; (**b**) the silique length of the control and *CclSBP6* transgenic *Arabidopsis*; (**c**) the statistics of silique length of the control and *CclSBP6* transgenic *Arabidopsis*; (**d**) the flower morphologies of the control and *CclSBP6* transgenic *Arabidopsis*; (**e**) the leaf morphologies of the control and *CclSBP6* transgenic *Arabidopsis*; (**f**) phenotypes of root length of the control and *CclSBP6* transgenic *Arabidopsis*; (**g**) PCR identification of transgenic *Arabidopsis*, wild type *Arabidopsis* as a negative control; OE4, OE14, and OE46 are the *CclSBP6* transgenic lines; (**h**) times to flowering of the T_3_ plants of three independent transgenic lines from *CclSBP6*; (**i**) number of leaves to flowering of the T_3_ plants of three independent transgenic lines from *CclSBP6*; (**j**) transcript levels of endogenous flowering related genes in the control and transgenic *Arabidopsis*.

*CclSBP6* did not exhibit homology with SPL gene family members of *Arabidopsis* (Figure 3b), and we speculated that this gene may perform special functions during the growth and development of citrus. Therefore, the function of *CclSBP6* was investigated by introduction into *Arabidopsis* (Figure 7). Twenty-three transgenic plants were obtained in the T_1_ generation; where all of the lines flowered later than their wild-type counterparts. For further analysis of the *CclSBP6* function, three independent transgenic lines in the third generation were selected for phenotypic observation (Figure 7g). Three *CclSBP6* transgenic lines flowered significantly later than the wild-type plants in terms of both days to flowering and the number of leaves. The average time to flowering of the transgenic plants ranged from 35.8 to 38.4 days, while that of the wild-type plants was 28.2 days (Figure 7h). The average number of leaves at flowering ranged from 16.0 to 16.8 in the transgenic plants, and was 11.8 in the wild-type plants (Figure 7g). In addition, the transgenic plants of *CclSBP6* showed multiple morphological changes, such as smaller flowers, slender leaves, shorter siliques, and extended root systems under long-days (Figure 7c–f).

To further elucidate the physiological functions of *CclSBP6* during the flowering of transgenic *Arabidopsis*, we analyzed the abundance of *Arabidopsis* early flowering related genes at 1-cm inflorescence stages of the wild-type. The levels of *Arabidopsis* endogenous *FLOWERING LOCUS T* (*FT*) and *SPL2/3/4/5/9* transcripts were clearly reduced in the transgenic lines (Figure 7j). These data suggested that *CclSBP6* functions may act as a floral repressor and might be involved in the citrus flowering. Previously, the transgenic *Arabidopsis* of citrus *miR156a* was developed [53]. The results showed that over-expression of citrus *miR156a* resulted in an extended juvenile phase in transgenic plants compared to the control plants. The phenotypes of *CclSBP6* and citrus *miR156a* transgenic plants were the same by genetic transformation of the *Arabidopsis*, where we speculated that *CclSBP6* may not be the target gene of *miR156a* in citrus.

### 3.8. Alternative Splicing and Functional Analysis of CclSBP7 in Transgenic Arabidopsis

**Figure 8:**
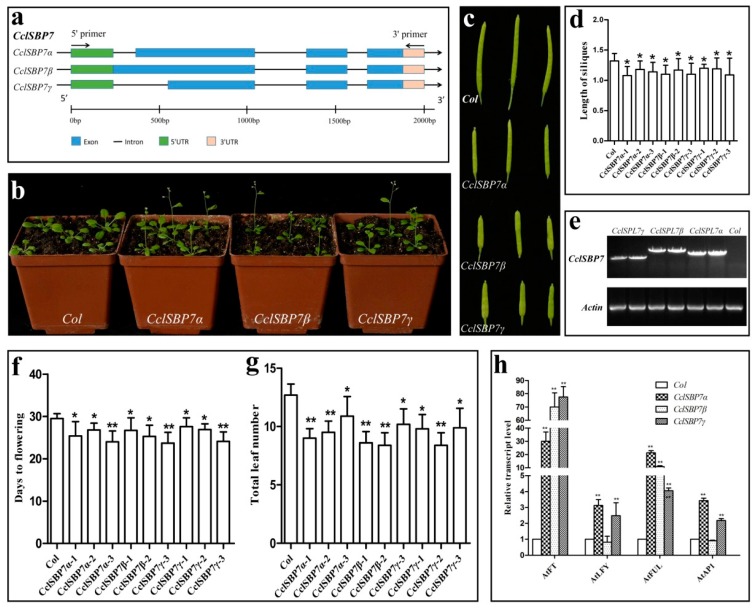
The gene structure of *CclSBP7* and phenotypes of transgenic *Arabidopsis* with *CclSBP7* under long-day conditions. (**a**) Schematic representation of the alternative processing of *CclSBP7*; rectangular boxes indicate exons and lines indicate introns; (**b**) phenotypes of transgenic *Arabidopsis* with *CclSBP7*; (**c**) the silique length of the control and *CclSBP7* transgenic *Arabidopsis*; (**d**) the statistical analysis of silique length transgenic *Arabidopsis* and the control; (**e**) PCR identification of transgenic *Arabidopsis*, wild-type *Arabidopsis* as a negative control; (**f)** times to flowering of the T_3_ plants of nine independent transgenic lines from three alternatively spliced transcripts of *CclSBP7*; (**g**) number of leaves to flowering of the T_3_ plants of nine independent transgenic lines from three alternatively spliced transcripts of *CclSBP7*; (**h**) transcript levels of the endogenous flowering gene in transgenic *Arabidopsis* and the control.

Further expression analysis of the *CclSBP7* was analyzed using RT-PCR, with the primers designed according to the open reading frame (ORF) of the *CclSBP7* from the citrus database, where they were named *CclSBP7α*. The templates from the adult plants’ mRNA were prepared. However, three bands were discovered in the RT-PCR analyses observation (Figure 1c). These amplification products were recovered and sequenced. Other than *CclSBP7α*, two different transcripts were also isolated. During comparisons of these cDNA sequences with each other, three transcripts of *CclSBP7* showed high identities with each other. They encoded the same 5′- and 3′-UTRs, whereas a region of these different transcripts was strongly divergent from the transcription initiation site (TSS) to 739 in the ORF through some nucleotide acids deletion and insertion. To further investigate whether these different transcripts were due to alternative splicing or they were transcripts from different genes, we isolated the full-length DNA of *CclSBP7*. Sequence analysis revealed that these *CclSBP7* transcripts came from the same genomic DNA, which was about 2 kb in size and had three introns and three exons with reference to the nucleotide sequence of *CclSBP7α*. *CclSBP7β* contained 1098 nucleotides of an ORF because of the first intron retention. *CclSBP7γ* contained 651 nucleotides of an ORF because of the partial deletion of the first exon (Figure 8a). The three unique transcripts of *CclSBP7* were conceptually translated and they showed three unique peptide sequences, and the three sequences were *CclSBP7α* (329aa), *CclSBP7β* (365aa), and *CclSBP7γ* (216aa).

To further analyze the function of the *CclSBP7*, three transgenic lines were randomly selected for each alternatively spliced transcript (Figure 8e). We selected 15 T_3_ plants for each transgenic line. Compared with control plants, the *CclSBP7α*, *CclSBP7β*, and *CclSBP7γ* transgenic lines flowered significantly earlier than the control plants in terms of both the number of leaves and days to flowering (Figure 8b). In the *CclSBP7α*, *CclSBP7β* and *CclSBP7γ*, the average time to flowering ranged from 23.7 to 27.6 days in six transgenic lines, whereas that of the control plants was 29.5 days (Figure 8f). The average number of leaves at flowering ranged from 8.4 to 10.9, and it was 12.7 in the control plants (Figure 8g). In addition, it is worth noting that the transgenic siliques were approximately 80% as long as the siliques of the control plants (Figure 8c–d). To evaluate the possible relation between the expression of *CclSBP7* and the early flowering phenotype of the transgenic *Arabidopsis*, the expression of some endogenous flowering-related genes from *Arabidopsis* was also assessed at 1-cm inflorescence stages of transgenic *Arabidopsis*. The levels of *FT*, *FRUITFULL* (*FUL*), *APETALA1* (*AP1*) and *LEAFY* (*LFY*) transcripts were clearly elevated in the transgenic plants compared to the wild-type (Figure 8h). These findings further supported our conclusion that the early flowering phenotype of the transgenic *Arabidopsis* was attributable to the expression of *CclSBP7*. Meanwhile, these data suggested that *CclSBP7* functions may act as a floral activator and might be involved in citrus flowering.

## 4. Discussion

The SBP-box genes are plant-specific transcription factors encoding proteins that contain a highly conserved SBP domain. This can specifically bind to the promoters of the floral meristem identity gene and it plays significant regulatory roles in plant growth and development, including sporogenesis, leaf development, vegetative and reproductive phase transitions, response to copper and fungal toxins and hormone signaling [4,5,15,21,22,23,25,54]. In a previous study, Shalom et al. identified the members of the SBP gene family in citrus, and they studied their seasonal expression patterns in buds and leaves, and in response to de-fruiting [30]. In this study, a comprehensive overview of the SBP-box gene family was undertaken, including the gene structures, phylogeny, chromosome locations, conserved motifs, and *cis*-elements in the promoter sequences as compared with the previous study. Meanwhile, we also performed a functional analysis of some of the members of the *SBP* gene family in *Arabidopsis*. The roles of SPLs and *miR156* as regulators of flowering have been extensively studied in *Arabidopsis* [15,17,24]. However, considerably less research has been done with woody plants. In the current work, similar to other plants, about two-thirds of the *CclSBP* contained sequences that were complementary to miR156. Furthermore, three (*CclSBP1*, *CclSBP2*, and *CclSBP3*) of 10 citrus SBP-box genes contained sequences that were complementary to *miR156* in the 3’-UTR region, except for the seven other members contained in the miR156-binding site located in the exon region, which comprised a relatively short protein size, consistent with previous reports on *Arabidopsis* [15,55]. The results provided a basis for elucidating the functions of the SBP-box genes in citrus.

It is believed that the SBP-box gene family has undergone gene duplications in many plants, such as *Arabidopsis* [55], rice [8], maize [9], *Populus* [10], grape [13], and apple [12]. The number of SBP-box genes identified in citrus was less than in *Arabidopsis*, which is inconsistent with the three-fold larger genome size of the *Citrus clementina* (367 Mb) versus that of *Arabidopsis* (125 Mb). This finding was similar to the results of a previous study that analyzed the CCCH gene family [51]. Evidence suggests that citrus is rather ancient and has an infrequent reproductive cycle in some taxa due to apomixis, male or female sterility, long juvenility and vegetative propagation [56]. These properties may greatly contribute to the restriction of genome expansion and evolution. Likewise, previous studies have demonstrated that there were no whole genome duplication events (WGDs) in citrus except an ancient triplication, called the γ event, which was shared by all the core eudicots [56]. There are, however, additional recent WGDs that have occurred in *Arabidopsis*, rice, maize, *Populus* and apple. Therefore, recent WGDs are likely to be the reason for a larger SBP-box gene number in these plants. It is noteworthy that *CclSBP6* and *CclSBP7* tended to be putative tandem duplicated genes based on the comprehensive analysis of gene locations and sequence properties. Several SBP-box gene pairs in citrus shared high-sequence similarity and were likely to be putative segmental duplicated genes (*CclSBP4*/*9*, *CclSBP8*/*10* and *CclSBP5*/*14*). The phylogenetic tree demonstrated that most citrus SBP-box genes were clustered more tightly with *Populus* and *Arabidopsis* rather than rice SBP-box genes. This is consistent with the fact that citrus, *Populus* and *Arabidopsis* are dicots and diverged more recently from a common ancestor than plants from the lineage leading to monocots. These results indicate that although plant SBP-box genes may be derived from a common ancestor and appeared after the divergence of plants and animals, many of them have undergone distinct patterns of differentiation and played different roles after the separation of each lineage.

To further reveal the possible roles of *CclSBP* genes, we constructed a phylogenetic tree based on 15 *CclSBPs*, 16 *AtSPLs*, and several SBP-box genes, where the functions have been characterized in other plant species (Figure S1). The phylogenetic tree was further divided into eight groups, and the members where functions have been characterized in each group were annotated using different colored circles. Within group I, aside from the miR156-binding site unknown of *AmSBP1*, all the other members were contained in the miR156-binding site. *AmSBP1* and *AtSPL3* have also been reported to bind *cis*-elements in the promoters of the floral organ identity genes *SQUAMOSA* and *APETALA1*(*AP1*), respectively [4], and *AtSPL3/4/5*, as well as *AmSBP1*, have all been implicated in the vegetative phase change and floral induction [4,18]. Another SBP-box gene within this group, *CNR*, has been reported to be pivotal for normal fruit ripening in tomato [27]. Surprisingly, a recent study has shown that the *CclSBP1* was able to promote flowering independently of the photoperiod in *Arabidopsis*, while *miR156* repressed its flowering-promoting activity [30]. Meanwhile, *CclSBP1*, *CclSBP2*, and *CclSBP3* have a close relationship with these functional investigated genes, and they showed high expression levels in flowering. These results suggest strongly that *CclSBP1*, *CclSBP2*, and *CclSBP3* have putative significant roles in the citrus flower and/or fruit development.

By contrast with Group I, the members of Group II were relatively large and lacked negative regulation by *miR156*. Only *PpSBP2* and *AtSPL14* were functionally characterized within this group. Although *PpSBP2* plays important roles in the regulation of copper homeostasis in *Barbula unguiculata* [57], it showed a relatively more distant relationship with other members from citrus and *Arabidopsis* compared to *AtSPL14*. *AtSPL14* has been reported to play significant roles in plant architecture [25]. As the ortholog gene of *AtSPL14*, *CclSBP12* probably has a similar function among them. Within group III, all the SBP-box members contained the miR156-binding site. *CclSBP6*, *CclSBP13*, *AtSPL9*, *AtSPL15*, and *OsSPL14* were clustered in one subgroup close together. In *Arabidopsis*, *AtSPL9* and *AtSPL15* play redundant roles in vegetative phase change and reproductive transition [20]. In rice, *OsSPL14* can repress vegetative branching and promote inflorescence branching [26]. Recently, new SBP-box genes (*NbSPL6*) have been identified from *Nicotiana benthamiana*, which is essential for the N-mediated resistance to the Tobacco mosaic virus. Similarly, *AtSPL6* functions in resistance to the bacterial pathogen *Pseudomonas syringae*, expressing the AvrRps4 effector [58]. Therefore, *CclSBP4* and *CclSBP9* may have similar function because of their closely phylogenetic relationship in the other subgroup.

Although they lack regulation by *miR156*, the members of group IV were characterized by functional diversity. Two SBP-box genes, *PpSBP1* and *PpSBP4*, were divided into one subgroup and were involved in regulating phase change and the circadian clock in moss [59]. In the other subgroup, *CclSBP7*, *AtSPL8*, *OsSPL8*, and *ZmLG1* were clustered closely together. The *AtSPL8* was the first SBP-box gene to be functionally characterized in *Arabidopsis* [22]. Although mutations of *AtSPL8* did not play roles in phase change, they have a profound effect on the seed set, petal trichome production, root growth, and male fertility [22,23,24]. In our study, the sequence analysis of several clones from *CclSBP7* obtained using RT-PCR resulted in the discovery of three alternative splicing transcripts. Transgenic *Arabidopsis* over-expressing these transcripts flowered earlier than the control. A previous study indicated that *CclSBP7* played a role in the floral inductive water deficit process. These results further suggested that *CclSBP7* acts as a floral inducer in citrus. One possible explanation for this observation is that the regulatory mechanism of *CclSBP7* differs between *Arabidopsis* and woody plants. Within group V, *CclSBP8* and *CclSBP10* were highly expressed in the leaf and homologous to *AtSPL13*, *teosinte glume architecture 1* (*tga1*), and *OsSPL6*. *AtSPL13* has been shown to affect the initiation of the first true leaves [54], maize *tga1* is involved in the ear glume development [60], and *OsSPL16* controls the grain size, shape, and quality in rice [61]. Therefore, we hypothesized that *CclSBP8* and *CclSBP10* may provide functions in controlling the characteristics of leaves and/or fruit.

Surprisingly, only *CclSBP11* was classified together with *AtSPL2*, *AtSPL10*, and *AtSPL11* into Group VI. *AtSPL10/11/2* are involved in the development of lateral organs, shape of the cauline leaves, and the number of trichomes on cauline leaves and flowers [19]. The results from spatial expression showed that *CclSBP11* was expressed at relatively higher levels in the leaves. Therefore, the *CclSBP11* probably had similar functions with the three *Arabidopsis* SBP-box genes in citrus. Within group VII, the *AtSPL7* and its ortholog gene *CclSBP15* were characterized by their large size and lack of miR156-binding site. Evidence reveals that *AtSPL7* can bind to the Cu-response element (CuRE) and be involved in copper homeostasis [62]. Furthermore, the *COPPER RESPONSE REGULATOR 1* (*CRR1*) in *Chlamydomonas reinhardtii* is the only one classified in group VIII, and it is homologous to *AtSPL7*. Similar to *AtSPL7*, *CRR1* recognizes and binds to the GTAC core sequence of CuRE in *Chlamydomonas reinhardtii*, and it has a similar function as the copper-responsive gene [7]. In addition, *CclSBP15* also exhibited responsiveness to abiotic stress, being up-regulated by drought. The results strongly suggest that *CclSBP15* probably has a similar function involved in copper homeostasis. The phylogenetic tree helped to predict the putative functions of the *CclSBP* genes based on the functions of SBP-box proteins in other species clustered in the same group. However, these findings only provide helpful information for understanding the function and regulation mechanism of *CclSBPs* in citrus. Further efforts will be exerted to find more direct evidence, including ectopic expression of different transcripts by transformation in citrus.

## 5. Conclusions

Function analysis has shown that SBP-box genes play crucial roles in the regulation of plant growth and development, especially in terms of flowering time, meristem identity and architecture, and fruit development. *Citrus*, as a perennial fruit tree, has distinct growth habits and quantitative traits in contrast to annual plant species. Comparative analysis of the SBP-box genes among various plants suggests that *CclSBPs* probably plays some unique roles and has undergone distinct evolutionary processes. In the present work, a total of 15 *CclSBPs* were identified from the whole genome sequence of *Citrus*, and the complete cDNA of all the members were isolated. Subsequently, a clear nomenclature was provided for them based on their chromosomal locations. Comprehensive analysis including gene structures, phylogenetic relationships, conserved motifs, expression patterns and *miR156*-mediated transcriptional regulation was conducted. We also performed a functional analysis of some *CclSBPs* in *Arabidopsis*. The results showed that it was able to change the flowering time of transgenic plants. The results provide useful information on *CclSBPs*, which should facilitate further research on elucidating the functions of SBP-box genes in citrus.

## Figures and Tables

**Table 1:** The feature of the SQUAMOSA-promoter binding protein (SBP)-box gene family in citrus.

Gene Name	Gene Locus	Genomic (bp)	CDs (bp)	No. of Transcript	EST Match	*miR156* Target
*CclSBP1*	Ciclev10009879m	1247	393	1	+^a^	3′-UTR
*CclSBP2*	Ciclev10016841m	2716	570	2	+^a^	3′-UTR
*CclSBP3*	Ciclev10017104m	1265	432	1	+^b^	3′-UTR
*CclSBP4*	Ciclev10019546m	3662	1674	2	+^a^	exon
*CclSBP5*	Ciclev10018697m	6422	2967	1	+^c^	no
*CclSBP6*	Ciclev10020532m	4055	1170	2	+^a^	exon
*CclSBP7*	Ciclev10021106m	2387	987	3	+^a^	no
*CclSBP8*	Ciclev10031834m	3660	1140	1	+^a^	exon
*CclSBP9*	Ciclev10031270m	5184	1536	2	+^a^	exon
*CclSBP10*	Ciclev10032171m	3272	939	1	+^b^	exon
*CclSBP11*	Ciclev10031391m	5362	1443	8	+^a^	exon
*CclSBP12*	Ciclev10000100m	5217	3309	1	+^a^	no
*CclSBP13*	Ciclev10011938m	2986	1167	2	+^a^	exon
*CclSBP14*	Ciclev10004227m	6489	3117	6	+^a^	no
*CclSBP15*	Ciclev10004348m	7069	2397	2	+^a^	no

Note: The result of the expressed sequence tags (EST) match comes from the HarvEST BLAST(Basic Local Alignment Search Tool) Search on NCBI (National Center for Biotechnology Information, Citrus C52 relaxed), +^a^ represents the percentage of EST match more than 95%, +^b^ represents the percentage match between 80–90%, +^c^ represents the percentage match lower than 80%.

## Supplementary Materials

The supplementary materials are available online at https://www.mdpi.com/2218-273X/9/2/66/s1. Figure S1. Functional diversity analysis of SBP-box genes in other plants, and phylogenetic relationship with citrus SBP-box genes. Phylogeny of SBP-box genes based on an alignment of the SBP-box domain, only genes for which functional data were available selected from other plant species. Green circles, presence; white circles, absence; black circles, unknown. Table S1 Gene-specific primers used in this study. Table S2. *Cis*-regulatory elements predicted in promoter region of *CclSBPs*.

## References

[1] Chen X., Zhang Z., Liu D., Kai Z., Li A., Long M. (2010). SQUAMOSA Promoter-Binding Protein-Like transcription factors: Star players for plant growth and development. J. Integr. Plant Biol..

[2] Yamasaki K., Kigawa T., Inoue M., Tateno M., Yamasaki T., Yabuki T., Aoki M., Seki E., Matsuda T., Nunokawa E. (2004). A novel zinc-binding motif revealed by solution structures of DNA-binding domains of *Arabidopsis* SBP-family transcription factors. J. Mol. Biol..

[3] Birkenbihl R.P., Jach G., Saedler H., Huijser P. (2005). Functional dissection of the plant-specific SBP-domain: Overlap of the DNA-binding and nuclear localization domains. J. Mol. Biol..

[4] Klein J., Saedler H., Huijser P. (1996). A new family of DNA binding proteins includes putative transcriptional regulators of the *Antirrhinum majus* floral meristem identity gene SQUAMOSA. Mol. Gen. Genet..

[5] Cardon G., Höhmann S., Klein J., Nettesheim K., Saedler H., Huijser P. (1999). Molecular characterisation of the *Arabidopsis* SBP-box genes. Gene.

[6] Lännenpää M., Jänönen I., HölttäUori M., Gardemeister M., Porali I., Sopanen T. (2004). A new SBP-box gene BpSPL1 in silver birch (*Betula pendula*). Physiol. Plant..

[7] Kropat J., Tottey S., Birkenbihl R.P., Depege N., Huijser P., Merchant S. (2005). A regulator of nutritional copper signaling in *Chlamydomonas* is an SBP domain protein that recognizes the GTAC core of copper response element. Proc. Natl. Acad. Sci. USA.

[8] Xie K., Wu C., Xiong L. (2006). Genomic organization, differential expression, and interaction of SQUAMOSA promoter-binding-like transcription factors and microRNA156 in rice. Plant Physiol..

[9] Hultquist J.F., Dorweiler J.E. (2008). Feminized tassels of maize mop1 and ts1 mutants exhibit altered levels of miR156 and specific SBP-box genes. Planta.

[10] Li C., Lu S. (2014). Molecular characterization of the SPL gene family in *Populus trichocarpa*. BMC Plant Biol..

[11] Salinas M., Xing S., Höhmann S., Berndtgen R., Huijser P. (2012). Genomic organization, phylogenetic comparison and differential expression of the SBP-box family of transcription factors in tomato. Planta.

[12] Li J., Hou H., Li X., Jiang X., Yin X., Gao H., Zheng Y., Bassett C.L., Wang X. (2013). Genome-wide identification and analysis of the SBP-box family genes in apple (*Malus* × *domestica* Borkh.). Plant Physiol. Biochem..

[13] Hou H., Li J., Gao M., Singer S.D., Wang H., Mao L., Fei Z., Wang X. (2013). Genomic organization, phylogenetic comparison and differential expression of the SBP-Box family genes in grape. PLoS ONE.

[14] Schwab R., Palatnik J.F., Riester M., Schommer C., Schmid M., Weigel D. (2005). Specific effects of microRNAs on the plant transcriptome. Dev. Cell.

[15] Wu G., Poethig R.S. (2006). Temporal regulation of shoot development in *Arabidopsis thaliana* by miR56 and its target SPL3. Development.

[16] Wang J.-W., Czech B., Weigel D. (2009). miR156-regulated SPL transcription factors define an endogenous flowering pathway in *Arabidopsis thaliana*. Cell.

[17] Yu N., Cai W.J., Wang S., Shan C.M., Wang L.J., Chen X.Y. (2010). Temporal control of trichome distribution by microRNA156-targeted SPL genes in *Arabidopsis thaliana*. Plant Cell.

[18] Jung J.-H., Seo P.J., Kang S.K., Park C.-M. (2011). miR172 signals are incorporated into the miR156 signaling pathway at the SPL3/4/5 genes in *Arabidopsis* developmental transitions. Plant Mol. Biol..

[19] Shikata M., Koyama T., Mitsuda N., Ohmetakagi M. (2009). *Arabidopsis* SBP-Box genes SPL10, SPL11 and SPL2 control morphological change in association with shoot maturation in the reproductive phase. Plant Cell Physiol..

[20] Schwarz S., Grande A.V., Bujdoso N., Saedler H., Huijser P. (2008). The microRNA regulated SBP-box genes SPL9 and SPL15 control shoot maturation in *Arabidopsis*. Plant Mol. Biol..

[21] Yamasaki H., Hayashi M., Fukazawa M., Kobayashi Y., Shikanai T. (2009). SQUAMOSA Promoter Binding Protein-Like7 is a central regulator for copper homeostasis in *Arabidopsis*. Plant Cell.

[22] Unte U.S., Sorensen A.M., Pesaresi P., Gandikota M., Leister D., Saedler H., Huijser P. (2003). SPL8, an SBP-box gene that affects pollen sac development in *Arabidopsis*. Plant Cell.

[23] Zhang Y., Schwarz S., Saedler H., Huijser P. (2007). SPL8, a local regulator in a subset of gibberellin-mediated developmental processes in *Arabidopsis*. Plant Mol. Biol..

[24] Xing S., Salinas M., Höhmann S., Berndtgen R., Huijser P. (2010). miR156-targeted and nontargeted SBP-Box transcription factors act in concert to secure male fertility in *Arabidopsis*. Plant Cell.

[25] Stone J.M., Liang X., Nekl E.R., Stiers J.J. (2005). *Arabidopsis* AtSPL14, a plant-specific SBP-domain transcription factor, participates in plant development and sensitivity to fumonisin B1. Plant J..

[26] Jiao Y., Wang Y., Xue D., Wang J., Yan M., Liu G., Dong G., Zeng D., Lu Z., Zhu X. (2010). Regulation of OsSPL14 by OsmiR156 defines ideal plant architecture in rice. Nat. Genet..

[27] Manning K., Tör M., Poole M., Hong Y., Thompson A.J., King G.J., Giovannoni J.J., Seymour G.B. (2006). A naturally occurring epigenetic mutation in a gene encoding an SBP-box transcription factor inhibits tomato fruit ripening. Nat. Genet..

[28] Chuck G., Whipple C., Jackson D., Hake S. (2010). The maize SBP-box transcription factor encoded by tasselsheath4 regulates bract development and the establishment of meristem boundaries. Development.

[29] Liu M.Y., Wu X.M., Long J.M., Guo W.W. (2017). Genomic characterization of miR156 and SQUAMOSA promoter binding protein-like genes in sweet orange (*Citrus sinensis*). Plant Cell Tissue Organ Cult..

[30] Shalom L., Shlizerman L., Zur N., Doronfaigenboim A., Blumwald E., Sadka A. (2015). Molecular characterization of SQUAMOSA PROMOTER BINDING PROTEIN-LIKE (SPL) gene family from *Citrus* and the effect of fruit load on their expression. Front. Plant Sci..

[31] Long J.-M., Liu C.-Y., Feng M.-Q., Liu Y., Wu X.-M., Guo W.-W. (2018). miR156-SPL modules regulate induction of somatic embryogenesis in citrus callus. J. Exp. Bot..

[32] Li J.-X., Hou X.-J., Zhu J., Zhou J.-J., Huang H.-B., Yue J.-Q., Gao J.-Y., Du Y.-X., Hu C.-X., Hu C.-G. (2017). Identification of genes associated with lemon floral transition and flower development during floral inductive water deficits: A hypothetical model. Front. Plant Sci..

[33] Huala E., Dickerman A.W., Garcia-Hernandez M., Weems D., Reiser L., LaFond F., Hanley D., Kiphart D., Zhuang M., Huang W. (2001). The Arabidopsis Information Resource (TAIR): A comprehensive database and web-based information retrieval, analysis, and visualization system for a model plant. Nucleic Acids Res..

[34] Wu G.A., Prochnik S., Jenkins J., Salse J., Hellsten U., Murat F., Perrier X., Ruiz M., Scalabrin S., Terol J. (2014). Sequencing of diverse mandarin, pummelo and orange genomes reveals complex history of admixture during citrus domestication. Nat. Biotechnol..

[35] Finn R.D., Bateman A., Clements J., Coggill P., Eberhardt R.Y., Eddy S.R., Heger A., Hetherington K., Holm L., Mistry J. (2014). Pfam: The protein families database. Nucleic Acids Res..

[36] Thompson J.D., Gibson T.J., Higgins D.G. (2003). Multiple Sequence Alignment Using ClustalW and ClustalX. Curr. Protoc. Bioinform..

[37] Crooks G.E., Hon G., Chandonia J.M., Brenner S.E. (2004). WebLogo: a sequence logo generator. Genome Res.

[38] Tamura K., Filipski A., Peterson D., Stecher G., Kumar S. (2013). MEGA6: Molecular Evolutionary Genetics Analysis Version 6.0. Mol. Biol. Evol..

[39] Guo A.-Y., Zhu Q.-H., Chen X., Luo J.-C. (2007). GSDS: A gene structure display server. Yi Chuan.

[40] Letunic I., Doerks T., Bork P. (2015). SMART: Recent updates, new developments and status in 2015. Nucleic Acids Res..

[41] Bailey T.L., Williams N., Misleh C., Li W.W. (2006). MEME: Discovering and analyzing DNA and protein sequence motifs. Nucleic Acids Res..

[42] Sun L.-M., Ai X.-Y., Li W.-Y., Guo W.-W., Deng X.-X., Hu C.-G., Zhang J.-Z. (2012). Identification and comparative profiling of miRNAs in an early flowering mutant of trifoliate orange and its wild type by genome-wide deep sequencing. PLOS ONE.

[43] Dai X., Zhao P.X. (2011). psRNATarget: A plant small RNA target analysis server. Nucleic Acids Res..

[44] Benson G. (1999). Tandem repeats finder: A program to analyze DNA sequences. Nucleic Acids Res..

[45] Bedell J.A., Korf I., Gish W. (2000). MaskerAid: A performance enhancement to RepeatMasker. Bioinformatics.

[46] Temnykh S., DeClerck G., Lukashova A., Lipovich L., Cartinhour S., McCouch S. (2001). Computational and experimental analysis of microsatellites in rice (*Oryza sativa* L.): frequency, length variation, transposon associations, and genetic marker potential. Genome Res..

[47] Lescot M., Déhais P., Thijs G., Marchal K., Moreau Y., Van de Peer Y., Rouzé P., Rombauts S. (2002). PlantCARE, a database of plant cis-acting regulatory elements and a portal to tools for in silico analysis of promoter sequences. Nucleic Acids Res..

[48] Shannon P., Markiel A., Ozier O., Baliga N.S., Wang J.T., Ramage D., Amin N., Schwikowski B., Ideker T. (2003). Cytoscape: A software environment for integrated models of biomolecular interaction networks. Genome Res..

[49] Yu G., Wang L.G., Han Y., He Q.Y. (2012). clusterProfiler: An R package for comparing biological themes among gene clusters. Omics-A J. Integr. Biol..

[50] Luo W., Brouwer C. (2013). Pathview: An R/Bioconductor package for pathway-based data integration and visualization. Bioinformatics.

[51] Liu S., Khan M.R.G., Li Y., Zhang J., Hu C. (2014). Comprehensive analysis of CCCH-type zinc finger gene family in citrus (*Clementine mandarin*) by genome-wide characterization. Mol. Genet. Genom..

[52] Clough S.J., Bent A.F. (1998). Floral dip: A simplified method for Agrobacterium-mediated transformation of Arabidopsis thaliana. Plant J.

[53] Wang C.-Y., Liu S.-R., Zhang X.-Y., Ma Y.-J., Hu C.-G., Zhang J.-Z. (2017). Genome-wide screening and characterization of long non-coding RNAs involved in flowering development of trifoliate orange (*Poncirus trifoliata* L. Raf.). Sci. Rep..

[54] Martin R.C., Asahina M., Liu P.-P., Kristof J.R., Coppersmith J.L., Pluskota W.E., Bassel G.W., Goloviznina N.A., Nguyen T.T., Martinez-Andujar C. (2010). The regulation of post-germinative transition from the cotyledon- to vegetative-leaf stages by microRNA-targeted SQUAMOSA PROMOTER-BINDING PROTEIN LIKE13 in *Arabidopsis*. Seed Sci. Res..

[55] Guo A.-Y., Zhu Q.-H., Gu X., Ge S., Yang J., Luo J. (2008). Genome-wide identification and evolutionary analysis of the plant specific SBP-box transcription factor family. Gene.

[56] Xu Q., Chen L.-L., Ruan X., Chen D., Zhu A., Chen C., Bertrand D., Jiao W.-B., Hao B.-H., Lyon M.P. (2013). The draft genome of sweet orange (*Citrus sinensis*). Nat. Genet..

[57] Nagae M., Nakata M., Takahashi Y. (2008). Identification of negative *cis*-acting elements in response to copper in the chloroplastic iron superoxide dismutase gene of the moss *Barbula unguiculata*. Plant Physiol..

[58] Padmanabhan M.S., Ma S., Burch-Smith T.M., Czymmek K., Huijser P., Dinesh-Kumar S.P. (2013). Novel positive regulatory role for the SPL6 transcription factor in the N TIR-NB-LRR receptor-mediated plant innate immunity. PLoS Pathog..

[59] Riese M., Zobell O., Saedler H., Huijser P. (2008). SBP-domain transcription factors as possible effectors of cryptochrome-mediated blue light signalling in the moss *Physcomitrella patens*. Planta.

[60] Wang H., Nussbaum-Wagler T., Li B., Zhao Q., Vigouroux Y., Faller M., Bomblies K., Lukens L., Doebley J.F. (2005). The origin of the naked grains of maize. Nature.

[61] Wang S., Wu K., Yuan Q., Liu X., Liu Z., Lin X., Zeng R., Zhu H., Dong G., Qian Q. (2012). Control of grain size, shape and quality by OsSPL16 in rice. Nat. Genet..

[62] Zhang H., Zhao X., Li J., Cai H., Deng X.W., Li L. (2014). MicroRNA408 Is Critical for the HY5-SPL7 Gene Network That Mediates the Coordinated Response to Light and Copper. Plant Cell.

